# Partially Evoked Epithelial-Mesenchymal Transition (EMT) Is Associated with Increased TGFβ Signaling within Lesional Scleroderma Skin

**DOI:** 10.1371/journal.pone.0134092

**Published:** 2015-07-28

**Authors:** Joanna Nikitorowicz-Buniak, Christopher P. Denton, David Abraham, Richard Stratton

**Affiliations:** Centre for Rheumatology and Connective Tissue Diseases, Research Department of Inflammation, Division of Medicine, UCL-Medical School, Rowland Hill Street, London, NW3 2PF, United Kingdom; Medical University of South Carolina, UNITED STATES

## Abstract

The origin of myofibroblasts in fibrotic conditions remains unknown and in systemic sclerosis (SSc) it has been proposed that activation of local fibroblasts, trans-differentiation of perivascular or vascular cells, recruitment of fibrocyte progenitors, or epithelial to mesenchymal transition (EMT) could be contributing. Data from our laboratory indicate that the epidermis in scleroderma is activated with the keratinocytes exhibiting a phenotype normally associated with tissue repair, including phosphorylation profiles indicative of TGFβ signaling. Since TGFβ is a known inducer of EMT, we investigated if there is evidence of this process in the SSc epidermis. In order to validate antibodies and primers, EMT was modeled in HaCaT cells cultured in the presence of TGFβ1. Skin sections were stained with phosho-SMAD2/3, as well as with epithelial and mesenchymal markers. Moreover, mRNA levels of transcription factors associated with EMT were studied in epidermal blister sheets. We observed critical changes in the scleroderma epidermis; showing significantly increased nuclear translocation of phosphorylated Smad2/3, consistent with active TGFβ signaling in SSc keratinocytes. While profound EMT could be induced in keratinocytes *in vitro* with the appearance of SNAI1/2 and FSP-1, and an accompanying loss of E-cadherin, in the scleroderma skin active TGFβ signaling was accompanied by only partial EMT-like changes characterised by induction of SNAI1 alone and with no loss of E-cadherin. Together, our findings support a model of altered differentiation and TGFβ dependent activation of scleroderma epithelial cells leading to a partially evoked EMT like process in the fibrotic skin.

## Introduction

Scleroderma (systemic sclerosis, SSc) is a severe form of skin and organ fibrosis in which TGFβ induced gene expression in persistently activated myofibroblasts has a key role. The origin of myofibroblasts present in the fibrotic tissue seen in SSc is unclear, and the epithelial to mesenchymal transition (EMT) process is given as one of the possibilities for the generation of these cells. A link between EMT and lung fibrosis is well established [1,2], but in the case of skin fibrosis the importance of EMT is disputed. TGFβ signaling and persistent inflammation are well known EMT inducers [3]. Recently we have demonstrated increased levels of pro-inflammatory S100A9 in the SSc epidermis [4]. Reports of enhanced expression of other inflammatory markers in SSc skin were also published [5,6].

Enhanced TGFβ1 and TGFβ receptor expression and active TGFβ signaling in SSc skin is well documented [7,8]. TGFβ also induces expression of other pro-fibrotic factors such as CTGF, which we have previously shown to be overexpressed in the SSc epidermis [4]. Its induction is mediated by the TGF-β/Smad pathway, which can also stimulate epithelial cells to undergo EMT and acquire the properties of mesenchymal cells [9]. This report explores whether EMT is a feature of the SSc epidermis and contributes to the increased population of myofibroblasts seen in the SSc skin.

## Materials and Methods

### Clinical materials

All clinical investigation must have been conducted according to the principles expressed in the Declaration of Helsinki. All subjects gave written informed consent to participate in this study. Ethical approval for the study was granted by the NRES Committee, London-Hampstead, Health Research Authority, Research Ethics Committee London Centre, reference 6398, “Elucidating the pathogenesis of systemic sclerosis by studying the skin, tissue and blood samples from patients and healthy volunteers. All SSc patients used in this report fulfilled the American College of Rheumatology (ACR) preliminary criteria for disease and were classified according to internationally accepted criteria [10], and so by definition also the new ACR-EULAR 2013 classification criteria [11]. All subjects gave written informed consent according to the Declaration of Helsinki to participate in this study, which was approved by the local Ethics Committee.

### Blister epithelial sheets

Suction blister cup (Ventipress, Upsala, Sweden) was applied to the forearm as previously described [12]. Under a pressure of 275–325 mmHg for 2 hours, 8mm blisters were raised in 9 healthy controls and 14 diffuse cutaneous subset SSc patients. The epidermal sheets (blister roof) were dissected, rinsed in PBS (Life Technologies, Glasgow, UK) and immediately placed in RNAlater (Life Technologies). Samples were then stored overnight at 4°C followed by longer-term storage at -70°C prior to RNA extraction.

### Cell culture

HaCaT cells (ATCC, Teddington, UK) were grown in 10% DMEM containing 10% FBS on 6 well plates. At approximately 80% confluence cells were washed twice in PBS, and serum-starved with 0.5% bovine serum albumin for 24 hours, prior to stimulation with recombinant TGF-β1 (R&D Systems, Minneapolis, US) at a concentration of 2ng/ml and 4ng/ml. Cells were monitored for change in morphology and phase-contrast photomicrographs were taken daily. Cell monolayers were lysed for mRNA, to assess expression of Snail1 and Snail2 at time points 0, 0.5, 1, 4, 6, 24, 48 and 72 hours.

The immunofluorescent staining and Western blotting assays were performed with HaCaT cells stimulated with 2ng/ml of TGFβ1 for 72 hours.

### Immunofluorescence

For histological studies samples from SSc patients (diffuse subset of less than 2 years duration) and healthy controls (both n = 6) were fixed in 4% paraformaldehyde and embedded in paraffin. 5μm thick sections were cut and deparaffinised in xylene, rehydrated in gradient concentrations of ethanol and water. Heat induced antigen retrieval was performed using sodium citrate buffer pH6 and unspecific binding was blocked with 10% normal serum. Sections were incubated with antibodies against Keratin14 (Vector Labs, Peterborough, UK), p-Smad2/3 (Santa Cruz, Heidelberg, Germany), vimentin (Santa Cruz), FSP-1 (Abcam, Cambridge, UK), E-cadherin (BD Bioscience, Oxford, UK), type IV collagen (Southern Biotech, Cambridge, UK), langerin (Abcam). Fluorophore-conjugated antibodies (Life Technologies) were used for detection. Finally, slides were washed and mounted. Appropriate concentrations of IgG (Vector) were used as primary antibody negative controls.

### Histological analysis

For each section, images were taken of at least 6 fields of view at x200 magnification. Imaging was done using Axioscope 2 MOTPlus and image analysis was performed on AxioVision 4.8 software (both from Zeiss Cambridge, UK).

### RNA isolation and quantitative RT-PCR

Total RNA was extracted using RNeasy kit (Qiagen, Manchester, UK) according to the manufacturer’s instructions and 1 μg/μl of total RNA was reverse transcribed using Quantitect (Qiagen). The cDNA template was then diluted 1 in 10 and used in qRT-PCR. Each 10μl reaction contained 5μl of SensiMix (Bioline, London, UK), 0.5μl of both forward and reverse primers (0.5μM), 2μl of nuclease free water and 2μl of cDNA template. The cycling conditions were as follows: initial denaturation at 95°C for 5 min followed by 40 cycles of amplification (denaturation at 95°C for 10s, annealing at 57°C for 20s, and extension for 10 s at 95°C).

Sequences of primers used in a study are listed below:

*TUBB* Forward: ACATACCTTGAGGCGAGCAA

           Reverse: TCACTGATCACCTCCCAGAA

*SNAI1* Forward: CAGGACTCTAATCCAGAGTTTACCT

           Reverse: ACAGAGTCCCAGATGAGCATTG

*SNAI2* Forward: GAACTGGACACACATACAGTGAT

           Reverse: GGTAGTCCACACAGTGATGG

Target transcript expression were normalised to the expression of the most stable housekeeping gene beta tubulin (TUBB) as previously described using the ΔΔCT method [13].

### Statistical analysis

The data was analysed using GraphPad Prism 5 (La Jolla, US) and Microsoft Excell (Redmond, US) software. The statistical significance was assessed by nonparametric Mann-Whitney test or student t-test. P≤ 0.05 was considered as statistically significant.

## Results

### Canonical TGFβ signaling in SSc skin

**Fig 1:**
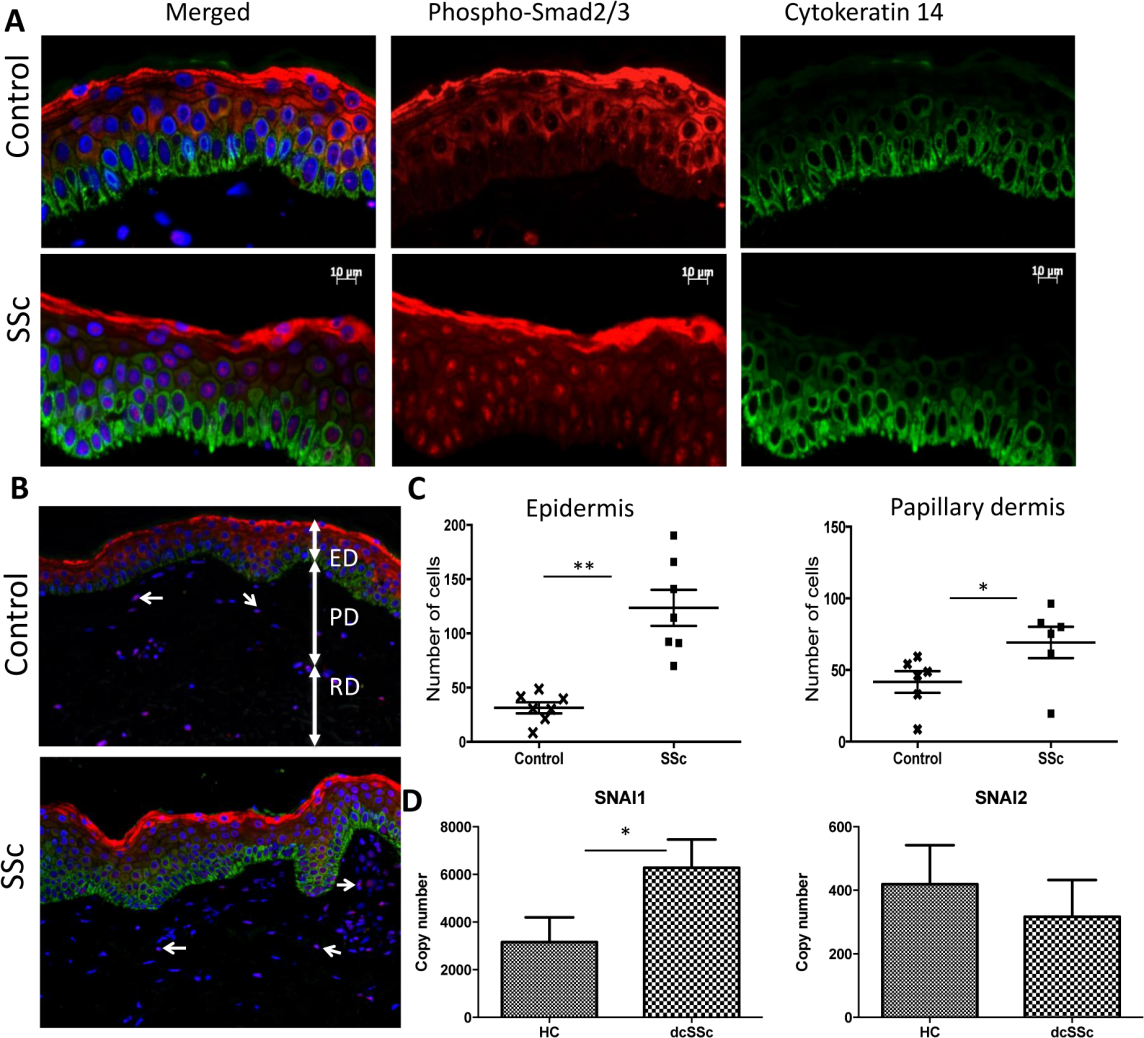
Canonical TGFβ signaling in SSc skin. (A) Representative images of double immunofluorescent staining performed to detect phospho-Smad2/3 (red) and K14 (green) in the epidermis of SSc patients and controls forearm skin sections (both n = 6, means of 5 high power views for each individual patient or control). DAPI (blue) was used to stain nuclei. Nuclear translocation of phospho-Smad2/3 was seen in SSc epidermal cells extending to suprabasal and granular layers. K14 expression was found to extend into suprabasal layers abnormally in SSc consistent with altered differentiation. (B) Also phospho-Smad2/3 nuclear translocation was seen in cells within the papillary dermis, increased in SSc sections (blue DAPI, red pSMAD2/3, double positive cells indicated with arrows). (C) When quantified the mean number of phospho-Smad2/3 positive cells was increased in SSc both in the epidermis (p<0.001) and in the adjacent papillary dermis (p<0.05) consistent with active TGFβ signaling. (D) qPCR of whole epidermal sheets obtained during suction blister formation revealed increased expression of SNAI1 transcription factor downstream of TGFβ. However SNAI2 was not increased. (ED = epidermis, PD = papillary dermis, RD = reticular dermis, ** = P<0.001, * = P<0.05).

In order to examine whether active TGFβ signaling was occurring Smad signalling in the SSc epidermis, was assessed by immunohistology. Analysis of sections stained with aniti-phospho-Smad2/3 antibodies revealed its co-localisation with nuclear stain DAPI (blue) in SSc skin (Fig 1). This nuclear expression was observed throughout the SSc epidermis, apart from the last granular layer, where the protein staining was in the cytoplasm. In contrast, in healthy epidermis no staining was observed in the basal cells, while the supra-basal cells showed cytoplasmic localisation. When quantified a pronounced difference was observed in the number of positive keratinocytes in the SSc epidermis (123.6±16.58) versus control (31.4±5.12) (p<0.005). In addition a significant (p<0.05) increase was noted in the number of p-Smad2/3 positive cells presumed fibroblasts in the SSc papillary dermis compared to controls (mean±SEM, 69.2±10.94 and 41.6±7.55 respectively). As we had described previously, keratinocyte differentiation was abnormally delayed in SSc with persistence of basal K14 into suprabasal layers.

### Modelling TGFβ-stimulated EMT *in vitro*

**Fig 2:**
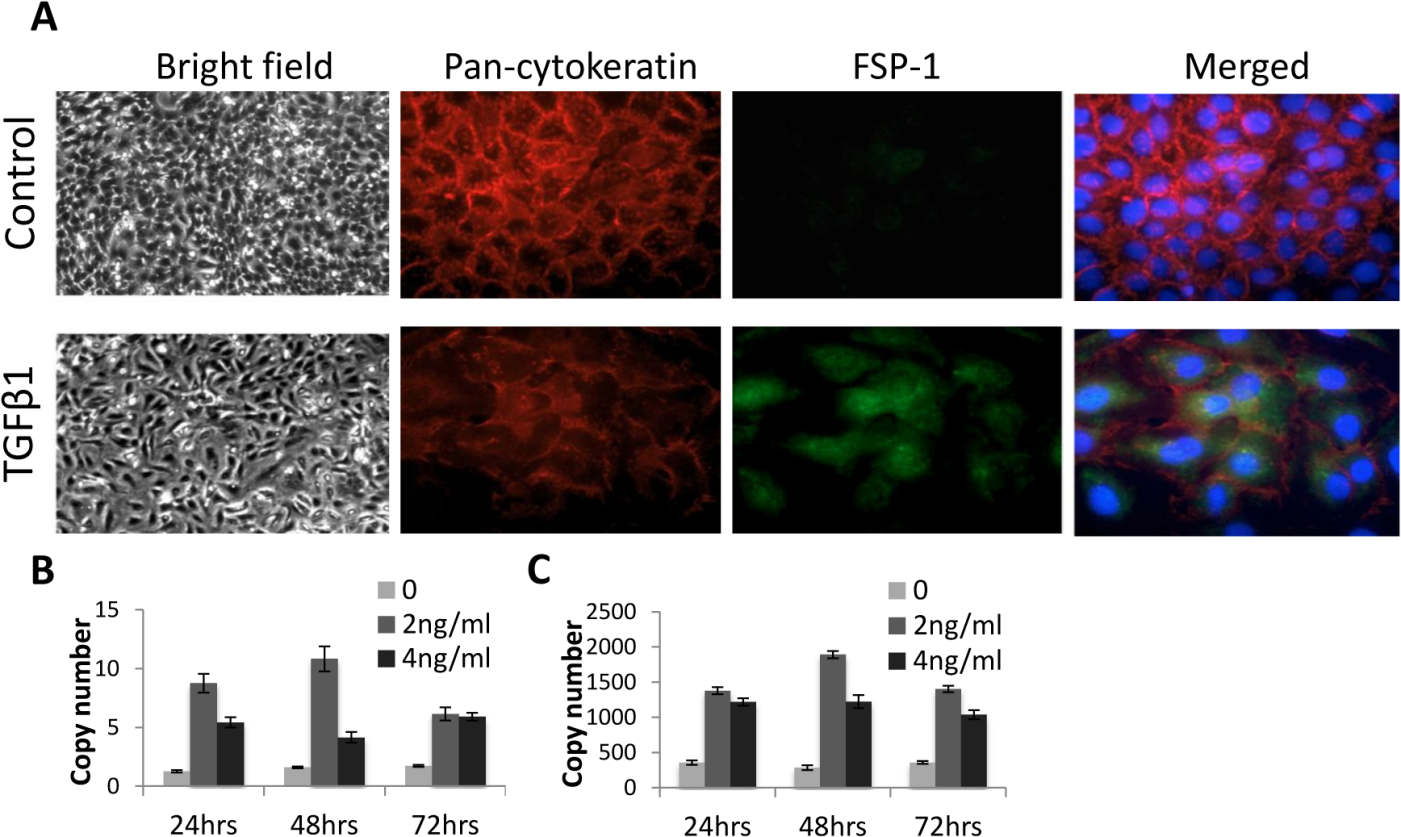
TGFβ stimulated EMT in HaCat cells. (A) HaCat cells cultured for 72hrs with TGFβ1 4ng/ml became elongated, lost cytokeratin and induced FSP-1 expression consistent with transition to a mesenchymal phenotype. (B) Culture with TGFβ1 also led to induction of both *SNAI1* and *SNAI2* mRNA maximal with TGFβ1 2 ng/ml and consistent with fully evoked EMT.

In an attempt to model EMT *in vitro* HaCaT cells were cultured with or without TGFβ 4ng/ml for 72 hours, leading to altered morphology, loss of cobblestone appearance, loss of E-cadherin and acquisition of FSP-1 (Fig 2) consistent with fully evoked EMT. Also consistent with this, mRNA for *SNAI1* and *SNAI2* were induced following treatment with TGFβ 2 and 4 ng/ml.

### Epithelial and mesenchymal markers in SSc skin

**Fig 3:**
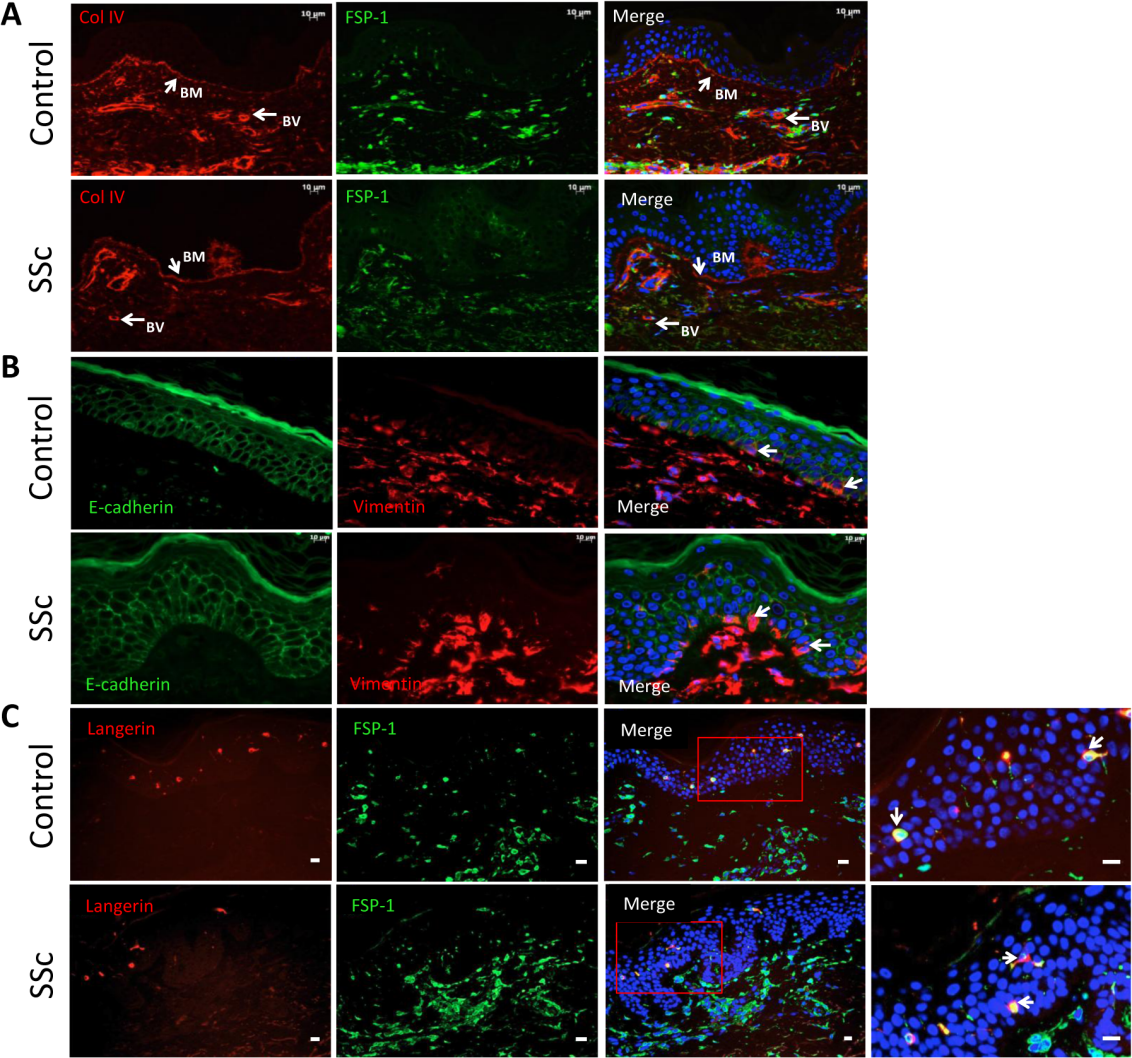
Epithelial and mesenchymal markers in SSc skin. (A) Representative images of double immunofluorescent staining of forearm skin sections performed to detect basement membrane protein collagen IV (red) and mesenchymal cell marker FSP-1 (green). The basement membrane collagen IV was not seen to be compromised in SSc, whereas some abnormal expression of FSP-1 in keratinocytes was seen in the SSc sections. (B) Additional stains for E-cadherin (green) and vimentin (red) were performed showing no loss of E-cadherin in SSc epidermal cells. Some expression of vimentin was however observed in the SSc epidermis. (C)Further analysis of FSP-1 positive cells was confirmed, since Langerhans cells within the epidermis are known to stain positive for mesenchymal markers. Immunostaining for FSP-1 (green) and Langerin (red) revealed that at least some of the FSP-1 positive cells were indeed from the Langerhans cell population (double positive cells shown with arrow). (BM = basement membrane, BV = blood vessels).

In order to begin to investigate whether an EMT like process is occurring in SSc sections of forearm skin were stained against the mesenchymal markers FSP-1 and vimentin, epithelial E-cadherin and the basement membrane protein collagen IV. The immunofluorescent staining results showed FSP-1 staining in control and SSc dermal cells presumed fibroblasts, and some positive staining cells in SSc epidermis but not controls. (Fig 3). However, collagen IV in the basement membrane of SSc patients did not appear compromised and the thickness of the basement membrane did not show any changes. There was also no indication of cells migrating through the basement membrane from the epidermis into the dermis. Also analysis of double staining of vimentin and E-cadherin did not reveal any differences in pattern or level of E-cadherin expression, between control and SSc skin sections. Vimentin staining was intense in the dermis of control and SSc. Some vimentin positive cells were detected in the SSc epidermis but not in controls.

A possible confounding factor is that Langerhans cells normally resident in the epidermis are known to express mesenchymal markers including FSP-1 and in order to address this possibility additional sections were stained with langerin (CD207) and FSP-1. The results of immunofluorescent staining of skin sections revealed that some but not allof the cells positive for FSP-1 were also positive for langerin confirming this as a possible confounding factor.

In addition, to determine whether active Smad dependent TGFβ signaling is stimulating SSc keratinocytes *in vivo* to undergo EMT, transcription factors critically associated with the transition, *SNAI1* and *SNAI2* were studied in the epidermal blister sheets [14,15]. The analysis revealed a 2-fold increase in number of *SNAI1* copies (p<0.05) in SSc compared to controls (6279±1182 and 3157±1038 respectively)(Fig 1). However, no significant differences were observed with *SNAI2* between SSc and control samples (SSc 370±128and control 419±122.7).

## Discussion

Previously we have identified that the epidermis in SSc skin lesions is highly activated, and capable of stimulating local fibroblasts as well as releasing pro-inflammatory such as S100A9 as well as pro-fibrotic CTGF [4]. Both proteins have been recently shown to be relevant to the EMT process and CTGF itself is considered in general to be a marker of activated mesenchymal cells [9,16]. In addition we have now shown that nuclear translocation of MRTF-A, a mechano-responsive transcription factor, is increased in the SSc epidermis, with MRTF-A itself providing a key signal in EMT [17,18]. Because of these findings we questioned whether EMT might be occurring in SSc and in this report, the effect of the disease microenvironment and active TGFβ signaling on potential EMT changes in SSc skin has been examined.

TGFβ ligand and downstream signaling pathways and mediators are strongly implicated in SSc pathogenesis, and TGFβ1 is well known to stimulate epithelial cells to undergo EMT *in vitro* [19]. As the origin of myofibroblasts present in fibrotic tissue in SSc and in general is unclear, it is plausible that EMT within the skin may contribute to generation of these cells. SSc epidermal keratinocytes losing their epithelial phenotype and gaining characteristics of mesenchymal cells could contribute to the increased population of local fibroblasts as previously suggested [20]. Moreover, reports suggesting EMT in SSc skin started to appear in recent years. Basal cells of the SSc epidermis with increased SFRP4, lacking caveolin-1 and with decreased E-cadherin expression along with the expression of vimentin were described [21]. Furthermore, Twist and Snail1 positive cells were found within eccrine glands of SSc patients [22]. Although interesting, the report was based on a limited number of SSc samples, and therefore might not be truly representative to the wider cohort of SSc patients.

A particular strength of the present study is the use of skin biopsies from well characterised patients to directly assess EMT in SSc skin. As anticipated, immunostaining revealed activated canonical TGF-β signaling in SSc epidermis as illustrated by increased nuclear staining for phosphorylated Smad2/3, and consistent with previous work by Dong et al (8). Possible origins of the enhanced TGFβ signaling include increased mechanical stress, altered integrin adhesion to and liberation of TGFβ from LAP.

Snail family proteins are direct targets of TGFβ in epithelial cells and have a critical role in EMT during development, in carcinogenesis, and in tissue repair [23]. Here we confirm induction of SNAI1 and SNAI2 during TGFβ induced EMT in vitro. Previous studies indicate that multiple signaling pathways synergise to induce Snail family members, and indicate that SNAI1 induction can occur independent of SNAI2 [24,25]. We found increased mRNA for SNA1 but not SNAI2 in the SSc epidermal tissue. Levels of SNAI1 and SNAI2 as analyzed in RNA extracted from epidermal blister sheets, showed a doubling of SNAI1 mRNA copy number in SSc compared to controls. Such an increase in gene expression, encoding an EMT-inducing transcription factor may suggest some differentiation towards an active EMT process in the SSc epidermis. Studies using human breast epithelial cells indicate a critical role for SNAI1 in the early phase of TGFβ induced EMT which is associated with induction of Vimentin but without profound loss of E-cadherin [26]. It is possible that changes seen in the SSc epidermis resemble this early partially evoked phase of EMT.

Downstream of TGFβ Snail proteins drive the wound healing response of keratinocytes by promoting migration and inflammation while suppressing terminal differentiation [27]. Over-expression of SNAI1 in mouse epidermis causes skin thickening and suprabasal expression of basal markers, consistent with a delay in terminal differentiation [25]. In fact we have shown similar changes in SSc previously and confirmed here, where delayed differentiation, and abnormal persistence of basal keratin K14, were shown by us to be features of SSc epidermis [4].

However, despite enhanced TGF-β signaling and induction of SNAI1, no loss of E-cadherin was observed in epidermal keratinocytes of SSc patients. In addition no decrease of collagen IV or loss of integrity of the basement membrane was observed in SSc which would be necessary for cells undergoing full invasive EMT to migrate into the dermis.

In both healthy control and diseased epidermis some cells positive for vimentin and E-cadherin were observed. Such co-expression of epithelial and mesenchymal markers could indicate EMT process or else indicate local Langerhans cells, which can express both markers [28].

One paradox is that we and others have shown that the epidermis is hyperproliferative in SSc, whereas TGFbeta is a known strong inhibitor of epithelial cell proliferation via Smad dependent effects on cylin dependent kinases and myc [4,29]. As clearly shown also in Whitfield’s article, proliferating Ki67 positive keratinocytes are seen in suprabasal layers in the disease epidermis which is a highly abnormal finding [30]. However mesenchymal cells can proliferate in response to TGFβ [31], and one possibility is that keratinocytes which have undergone partial transiton to mesenchymal cells in SSc are responding to TGFβ by proliferation.

Although, active Smad/TGFβ signaling and enhanced CTGF expression, along with enhanced levels of SNAI1 mRNA in SSc are suggestive of EMT induction, our results do not confirm full transition in the SSc epidermis *in vivo*. Therefore changes in the SSc epidermis might reflect partially evoked EMT as has already been reported during wound re-epithelialisation [32,33]. This may be relevant to the persistent fibrotic phase of SSc since a failure to fully transform might make the cells less amenable to switch off signals that normally terminate this process in physiological wound healing. This would be consistent with our earlier studies suggesting that a persistent wound healing phenotype is present in keratinocytes in SSc lesional skin. However, our present model is that the activated epidermis is promoting local fibroblasts by cross talk with dermal cells rather than through a full EMT process. The concept of partial EMT leading to fibrogenesis is novel and may have broader applicability in tissue repair or fibrosis. Our findings may also explain some of the contradictory previous studies concerning EMT in scleroderma tissue.

## Conclusions

SSc epidermis shows changes consistent with partially evoked form of EMT: active TGFβ signaling and increased SNAI1 mRNA. In SSc epidermal cells acquire some mesenchymal features and may contribute to fibrosis without fully transforming into a myofibroblast population. This might represent an important novel pathogenic cell type in SSc that warrants further investigation.

## Supporting Information

S1 Data S1 Data supporting the qPCR for HaCat cells induction of SNAI1&2, for qPCR analysis of epidermal sheets and for quantification of pSMAD2/3 positive keratinocytes and dermal fibroblasts are attached as Excel sheet. (XLSX)
